# Encoding surprise by retinal ganglion cells

**DOI:** 10.1371/journal.pcbi.1011965

**Published:** 2024-04-17

**Authors:** Danica Despotović, Corentin Joffrois, Olivier Marre, Matthew Chalk

**Affiliations:** Institut de la Vision, INSERM, CNRS, Sorbonne Université, Paris, France; Scuola Internazionale Superiore di Studi Avanzati, ITALY

## Abstract

The efficient coding hypothesis posits that early sensory neurons transmit maximal information about sensory stimuli, given internal constraints. A central prediction of this theory is that neurons should preferentially encode stimuli that are most surprising. Previous studies suggest this may be the case in early visual areas, where many neurons respond strongly to rare or surprising stimuli. For example, previous research showed that when presented with a rhythmic sequence of full-field flashes, many retinal ganglion cells (RGCs) respond strongly at the instance the flash sequence stops, and when another flash would be expected. This phenomenon is called the ‘omitted stimulus response’. However, it is not known whether the responses of these cells varies in a graded way depending on the level of stimulus surprise. To investigate this, we presented retinal neurons with extended sequences of stochastic flashes. With this stimulus, the surprise associated with a particular flash/silence, could be quantified analytically, and varied in a graded manner depending on the previous sequences of flashes and silences. Interestingly, we found that RGC responses could be well explained by a simple normative model, which described how they optimally combined their prior expectations and recent stimulus history, so as to encode surprise. Further, much of the diversity in RGC responses could be explained by the model, due to the different prior expectations that different neurons had about the stimulus statistics. These results suggest that even as early as the retina many cells encode surprise, relative to their own, internally generated expectations.

## Author summary

A long-standing theory suggests that sensory neurons work to transmit as much visual information as possible using little energy. According to this idea, sensory neurons should put most energy into encoding stimuli that are unexpected or surprising. Previous research has shown that this may be true for certain neurons in the retina, that respond strongly when they see something unusual, like a sudden change in a sequence of flashes. Here, to test this theory, we presented the retina with random sequences of light flashes, and measured whether retinal neuron responses correlated with how surprising each flash in the sequence was. Consistent with the theory, we found that retinal neuron responses in our experiment could be explained by a model that assumed that they encode surprise. Moreover, differences between each neuron’s responses could be predicted due to their different expectations about which stimuli were most likely.

## Introduction

Visual scenes are highly correlated, both in space and time. It has been hypothesized that neurons in early sensory areas have evolved to exploit this structure, by only encoding ‘surprising’ sensory signals, that cannot be predicted based on their spatio-temporal context. This efficient coding theory can account for many qualitative aspects of neural responses in early sensory areas, such as the stimulus selectivity of neurons in the retina [1–3], as well as primary visual [4–6] and auditory [7, 8] cortices.

A central prediction of the efficient coding theory is that neurons should best encode stimuli that are surprising, given the recent stimulus history. There appears to be some evidence for this in early visual and auditory areas, where neurons have been found that respond most strongly to rare or surprising stimuli [9, 10]. In the retina, previous studies found that when a sequence of full-field light flashes are presented, many neurons respond most strongly at the moment the sequence of flashes is stopped, and where another flash would be expected. This phenomenon was labelled the ‘omitted stimulus response’ (OSR), since it can be considered to be a response to the stimulus that was unexpectedly omitted [11].

However, if neurons in the retina really do encode surprise, then their responses should vary in a graded way as one varies the level of stimulus surprise. Unfortunately previous studies [11–13] typically measured neural responses to a limited range of flash sequences (e.g. repeated sequences of *n* consecutive flashes presented in a row). As a result, it is hard to conclude from these studies whether neurons in the retina encode surprise.

To address this question, we presented retinal ganglion cells (RGCs) with extended sequences of stochastically occurring full-field flashes. This extended stimulus included many different sequences of flashes and silences, while the degree of ‘surprise’ for each flash (or period of silence between flashes) could be quantified mathematically, depending on the previous sequence of flashes and silences. We could thus test how RGC responses varied with the level of surprise. Interestingly, we found that the responses of RGCs to these stimulus sequences could be well explained by a simple normative model, which described how neurons optimally combined their prior expectations about the stimulus with the recent stimulus history to encode surprise. Further, we found that much of the diversity in the responses of different recorded RGCs could be explained by this model, due to the different levels of ‘confidence’ that different neurons had in their prior expectations. Future work will be needed to elucidate the underlying neural mechanisms that underly these computations.

Our study provides support for the predictive coding model of retinal coding, while shedding light on the different prior expectations that different RGCs have about the environment. More generally, it shows that, already at the stage of the retina, many ganglion cells do not encode the physical stimulus itself, but how unexpected this stimulus is, with different prior expectations for different cells.

## Materials and methods

### Experimental setup

The recordings were performed in the axolotl retina, using a multi-electrode array with 252 electrodes with 60 *μ*m spacing (procedure described in detail in [14]). The experiment was performed in accordance with institutional animal care standards of Sorbonne Université. The raw signal, recorded at 20 kHz sampling rate, was high-pass filtered at 100 Hz and then sorted offline using SpyKing Circus software [15]. The stimulus consisted of full-field dark flashes. The reason for using dark flashes was the dominance of OFF type cells in axolotl. The dark flashes had a duration of 40 ms, with 80 ms period between the flashes, (∼12 Hz frequency) (as in [11]).

### Stimulus statistics

We generated sequences of flashes and silences (i.e. where no flash occured in a 120ms window) using a stochastic model. The number of flashes and periods of silent states presented in a row was drawn from a negative binomial distribution, with parameters *r* and *p*. In the case of flashes, we varied the first parameter, *p*, at 20 minute intervals between three different values (0.98, 0.8 and 0.01, consecutively). The second parameter, *r* was adjusted so as to maintain a constant mean, of 7 flashes presented in a row. The length of the silence sequence was drawn from a geometrical distribution with a fixed mean *p* (*p* = 9). Changing *p* alters the degree to which the distribution is clustered around the mean. However, we observed no difference in the neural responses recorded with different values of *p*. As a result we concatenated data from neural responses to all three stimulus distributions for the rest of our analysis.

### Data analysis

**Fig 1:**
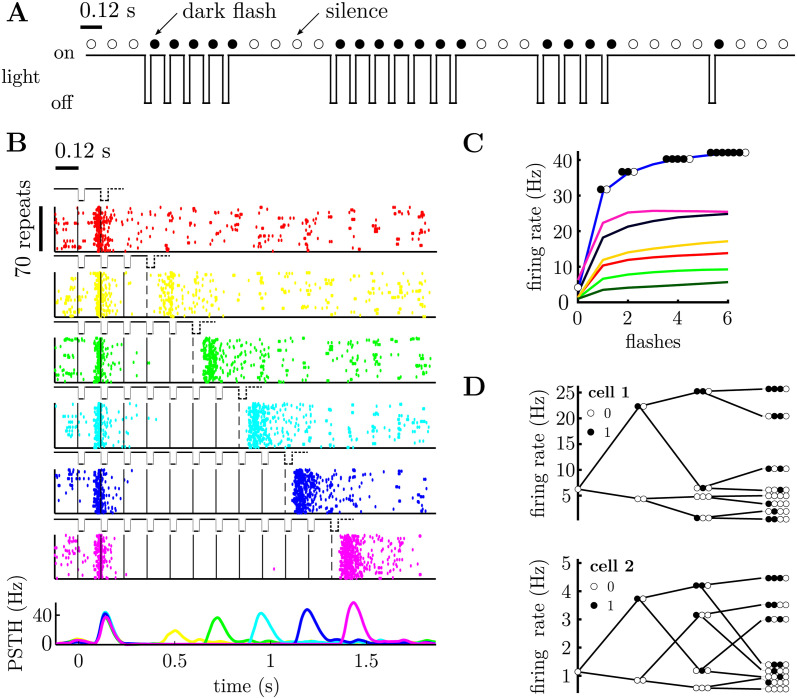
Retinal ganglion cells’ responses to a sequences of dark flashes and silences. **A.** Stimulus excerpt, showing periodic sequences of dark flashes. Each flash lasts 40 ms with 80 ms between. The 120 ms bin containing a single dark flash is marked with a filled circle. A bin without a flash (called a ‘silence’) is marked by an open circle. **B.** Raster plot for one cell. A solid vertical line marks the occurrence of each flash; a dashed line indicates an ‘omitted’ flash, following a sequence of flashes. Each raster plot shows the cell’s response to a different number of consecutive flashes (ranging from 1 to 11), with 70 repeats shown in each row of the raster. The bottom row shows the peri-stimulus time histogram (PSTH) for different numbers of consecutive flashes (colors denote the number of flashes). There is an increase in firing rate after the missing flash, called the omitted stimulus response (OSR). The OSR magnitude increases with the number of flashes. **C.** OSR for 7 cells, following a varying number of consecutive flashes (filled circles) followed by silence (open circles). **D.** Tree-plot, showing the mean response of two representative cells to different sequences of flashes (filled circles) and silences (empty circles). Cell 1 is the cell plotted in pink in panel C. Each column of the tree-plot shows the average response of the neuron to all stimulus sequences of a given length that end with silence (tree-plots corresponding to sequences ending with a flash are shown in S1 Fig). Moving right-ward the tree-plot branches out to include the effect of stimuli presented further in the past. The top branch of the tree-plot shows the cells’ responses to a series of consecutive flashes followed by silence, as in panel C. Other branches show the cells’ responses to all the different possible flash sequences of a given length.

To generate the spike raster plots shown in Fig 1, we aligned the spiking responses of neurons to a sequence of *n* flashes presented in a row. The peri-stimulus-time-histogram (PSTH) plotted in the bottom row of Fig 1 was computed by averaging the spike count recording over all the stimulus repeats, and then averaging over a 5 ms time bin.

For the remainder of the analysis, we discretised the neural responses and stimulus into time bins of length 120 ms (the time between consecutive flashes). The stimulus presented in each time-bin was treated as a binary variable: ‘1’ if there was a (dark) flash, ‘0’ otherwise. The average firing rate in each bin was computed by average the spike count over all repetitions of a stimulus sequence of length *n*. Except where stated explicitly in the text, we set *n* = 8 (so there were 256 distinct stimulus sequences used to compute the average firing rate).

### Neural model

For our model, we assumed that at each time-bin, *t*, neurons fire spikes drawn from a Poisson distribution with mean, λ_*t*_, given by:

$$\begin{array}{c} \lambda_{t}=f\left(as_{t}+b\right) \end{array}$$ (1)

where *s*_*t*_ is the encoded surprise at time *t*, *f* is a non-linearity, and *a* and *b* are parameters describing the gain and bias, respectively. The non-linearity, *f*(*x*) = log(1 + *e*^*x*^) (soft-ReLU), was kept fixed for all the cells.

The surprise at time *t* is defined as:

$$\begin{array}{c} s_{t}=-\text{log}\;p\left(x_{t}|x_{t-1},x_{t-2},\ldots ,\theta \right) \end{array}$$ (2)

where *p*(*x*_*t*_|*x*_*t*_, *x*_*t*−2_, …, *θ*) is the probability of observing no flash or a flash at time *t* (*x*_*t*_ = 0 or 1 respectively) given the stimulus *x* at previous times, and the internal model of the cell, parameterized by *θ*.

### Internal model

The computed surprise depends on each cell’s internal model of the stimulus statistics. We first considered a binary Markov model, where the probability of observing a flash at time *t* is assumed to depend only on whether a flash was observed in the previous time bin. This model has two parameters: *θ*_0_ = *p*(*x*_*t*_ = 1|*x*_*t*−1_ = 0), and *θ*_1_ = *p*(*x*_*t*_ = 1|*x*_*t*−1_ = 1). For the Markov 2 model, we simply extend the observed history to 2 previous states, yielding a total of 4 parameters: *θ*_0_ = *p*(*x*_*t*_ = 1|*x*_*t*−1_ = 0, *x*_*t*−2_ = 0), *θ*_1_ = *p*(*x*_*t*_ = 1|*x*_*t*−1_ = 1, *x*_*t*−2_ = 0), *θ*_2_ = *p*(*x*_*t*_ = 1|*x*_*t*−1_ = 0, *x*_*t*−2_ = 1), and *θ*_3_ = *p*(*x*_*t*_ = 1|*x*_*t*−1_ = 1, *x*_*t*−2_ = 1).

### Inferring the transition probabilities

Next, we considered an ‘adaptive belief model’ where the transition probabilities, *θ*_*i*_ = *p*(*x*_*t*_|*x*_*t*−1_ = *i*), are not known in advance, but must be inferred by combining each cell’s prior belief with new observations, using Bayes’ law, as follows:

$$\begin{array}{c} p(\theta |x_{t},x_{t-1},\ldots )\propto p(x_{t}|x_{t-1},\theta )p(\theta |x_{t-1},x_{t-2},\ldots ) \end{array}$$ (3)

where *p*(*x*_*t*_|*x*_*t*−1_, *θ*) is the likelihood of observing *x*_*t*_ given *x*_*t*−1_, and is described by a Bernoulli distribution:

$$\begin{array}{c} p(x_{t}|x_{t-1}=i,\theta_{i})=\theta_{i}^{x_{t}}{\left(1-\theta_{i}\right)}^{1-x_{t}}. \end{array}$$ (4)

Let us assume that at time *t* − 1, the posterior distribution over *θ*_*i*_, *p*(*θ*|*x*_*t*−1_, *x*_*t*−2_, …), is described by the beta distribution with parameters $\alpha_{t-1}^{i}$ and $\beta_{t-1}^{i}$:

$$\begin{array}{c} p(\theta_{i}|x_{t-1},x_{t-2},\ldots )\propto \theta^{\alpha_{t-1}^{i}-1}{\left(1-\theta \right)}^{\beta_{t-1}^{i}-1}. \end{array}$$ (5)

Now, at time *t*, multiplying this distribution by the likelihood according to Bayes law (Eq 7) will result in a new beta-distribution, with parameters:

$$\begin{array}{ccc} \alpha_{t}^{i} & \leftarrow & \alpha_{t}^{i}+x_{t}x_{t-1}^{i}{\left(1-x_{t-1}\right)}^{1-i} \end{array}$$ (6)

$$\begin{array}{ccc} \beta_{t}^{i} & \leftarrow & \beta_{t}^{i}+\left(1-x_{t}\right)x_{t-1}^{i}{\left(1-x_{t-1}\right)}^{1-i} \end{array}$$ (7)

The probability of observing *x*_*t*_ = 1 given previous observations is then given by:

$$\begin{array}{ccc} p(x_{t}=1|x_{t-1}=i,x_{t-2},x_{t-3},\ldots ) & = & \int_{\theta }p\left(x_{t}=1|x_{t-1}=i,\theta_{i}\right)p(\theta_{i}|x_{t-1},x_{t-2},\ldots ) \end{array}$$ (8)

$$\begin{array}{ccc} & = & \frac{\alpha_{t-1}^{i}}{\alpha_{t-1}^{i}+\beta_{t-1}^{i}} \end{array}$$ (9)

$$\begin{array}{ccc} & = & \frac{n_{i\to 1}}{n_{i\to 1}+n_{i\to 0}}, \end{array}$$ (10)

where *n*_*i*→0_ and *n*_*i*→1_ describe the number of occurrences of the transitions *i* → 0 and *i* → 1, respectively.

### Adaptive surprise model

The statistics of the external world are not static, but change in time. To take this into account, we could assume there is a non-zero probability of transition matrix changing between two observations (a ‘dynamic belief model’). Performing exact Bayesian inference in this case requires expensive numerical integration, which may be difficult to perform by individual neurons in the retina. However, in they found that such a dynamic belief model could be approximated by a ‘forgetful’ model, where recent observations are weighted more strongly than the ones in the past. In contrast to the optimal Bayesian model, their leaky integration model results in simple linear parameter updates, and could thus be easy to implement neurally.

In practice, we can implement the ‘forgetful’ model of Meyniel et al. [16], by modifying the update rules described earlier for *α*^*i*^ and *β*^*i*^ as follows:

$$\begin{array}{ccc} \alpha_{t}^{i} & \leftarrow & (1-\eta )\alpha_{t}^{i}+\eta \alpha_{0}^{i}+x_{t}x_{t-1}^{i}{\left(1-x_{t-1}\right)}^{1-i} \end{array}$$ (11)

$$\begin{array}{ccc} \beta_{t}^{i} & \leftarrow & (1-\eta )\beta_{t}^{i}+\eta \beta_{0}^{i}+\left(1-x_{t}\right)x_{t-1}^{i}{\left(1-x_{t-1}\right)}^{1-i} \end{array}$$ (12)

where 1 > *η* > 0 is a leak term that results in forgetting observations far in the past, while $\alpha_{0}^{i}$ and $\beta_{0}^{i}$ determine the steady state values of $\alpha_{t}^{i}$ and $\beta_{t}^{i}$ in the absence of new observations. With this update rule, the probability of observing a *x*_*t*_ = 1 given *x*_*t*−1_ = *i* is given by:

$$\begin{array}{c} p(x_{t}=1|x_{t-1}=i,x_{t-2},x_{t-3},\ldots )=\frac{{\tilde{n}}_{i\to 1}+\alpha_{0}^{i}}{{\tilde{n}}_{i\to 1}+{\tilde{n}}_{i\to 0}+\beta_{0}^{i}}, \end{array}$$ (13)

where ${\tilde{n}}_{i\to j}$ is the ‘effective’ number of observations of a transition *i* → *j*, after taking into account the leak, when *η* > 0:

$$\begin{array}{c} {\tilde{n}}_{i\to j}=\sum_{k=0}^{\infty }{\left(1-\eta \right)}^{k}x_{t-k}^{j}x_{t-1-k}^{i}{\left(1-x_{t-k}\right)}^{\left(1-j\right)}{\left(1-x_{t-1-k}\right)}^{\left(1-i\right)}. \end{array}$$ (14)

We assumed that the leak, *η* was the same for all cells. We used a value of *η* = 0.2. However, similar results were obtained when we increased or decreased the leak by a small amount.

In contrast, the parameters of the prior, $\alpha_{0}^{i}$ and $\beta_{0}^{i}$, were allowed to vary for different cells. This allowed us allowed to investigate how different cells’ ‘prior expectations’ for different transitions affected their responses. (Note that for notational simplicity we dropped the subscript ‘0’ in the main text.).

In S1 Text materials we describe a numerical implementation of the exact Bayesian inference model, which assumes that there is a constant probability that the transition probabilities of the Markov model change at each time-step. As mentioned, the results of this model were very similar to our approximate model, described above (S5 Fig).

### Model fitting

We fitted the internal model parameters (see previous section), and the gain and bias of the response curves (Eq 4) using Maximum Likelihood (ML) algorithm [17]. For this, we assumed a Poisson noise model, resulting in a log-likelihood:

$$\begin{array}{c} L=\sum_{t}n_{t}\;\text{log}\;f_{t}-f_{t} \end{array}$$ (15)

where *f*_*t*_ and *n*_*t*_ are the spike count predicted by the model and observed spike count at time *t*, respectively. All models were fitted using algorithms with multiple starting points (MultiStart in MATLAB, 50 starting points, random initial parameters).

The data analysis and model fitting were done in MATLAB R2021a. Code and data will be available upon paper acceptance.

## Results

### RGC responses to flash sequences

We used a multi-electrode array to record retinal ganglion cells (RGCs) of an axolotl. We presented a visual stimulus, consisting of random sequences of full-field dark flashes, interleaved with periods of silence (Fig 1A; see Methods: Stimulus statistics for details). Recorded neural activity was sorted into single unit responses using SpyKing Circus [15].

We were interested in neurons that exhibited an ‘omitted stimulus response’ (OSR), where they responded to the absence of a flash, following several flashes presented in a row [11]. We thus selected 48 out of 114 single unit responses for further analysis, that showed (i) high quality recording (quantified by low number (<1%) of refractory period violations, where refractory period is 2 ms), and (ii) the presence of an OSR (quantified as a peak around 120 ms after the omitted flash).

Fig 1B shows the example responses of one of these cells to a varying number of flashes presented in a row. As can be seen, this cell responded strongly to the first flash in a sequence, and shortly after the sequence had ended (i.e. the OSR). The size of the OSR increased monotonically with the number of flashes presented in a row.

For our analysis, we converted the stimulus to a binary variable, which was set to 1 or 0 depending on whether there was a dark flash (stim. = 1) or a period of silence (stim. = 0) within a given 120ms window. Neural responses were taken to be the number of spikes that occurred within each 120ms window.

To see how the OSR varied with the number of consecutive flashes, we computed the average response of each neuron, given a ‘stimulus history’ consisting of a varying number of consecutive flashes followed by silence (Fig 1C). The OSR increased monotonically with the number of flashes for all cells. However, we observed differences in the rate of increase as well as the maximum firing rate for different cells (Fig 1C).

Finally, to see how neural responses depended on all possible stimulus sequences (and not the number of consecutive flashes), we constructed ‘tree-plots’ (Fig 1D), showing each neuron’s average response to all possible stimulus sequences of a given length, ending with a silence (the complementary tree-plot, for sequences ending with a flash, is shown in S1 Fig). The top branch of this tree plot corresponds to the OSR, shown in Fig 1D. However, many cells that showed a qualitatively similar OSR (i.e. that increased with the number of consecutive flashes) exhibited very different tree-plots, identifying clear differences in how they responded to different patterns of flashes and silences (e.g. Fig 1D).

### Modeling ‘surprise encoding’ by RGCs

**Fig 2:**
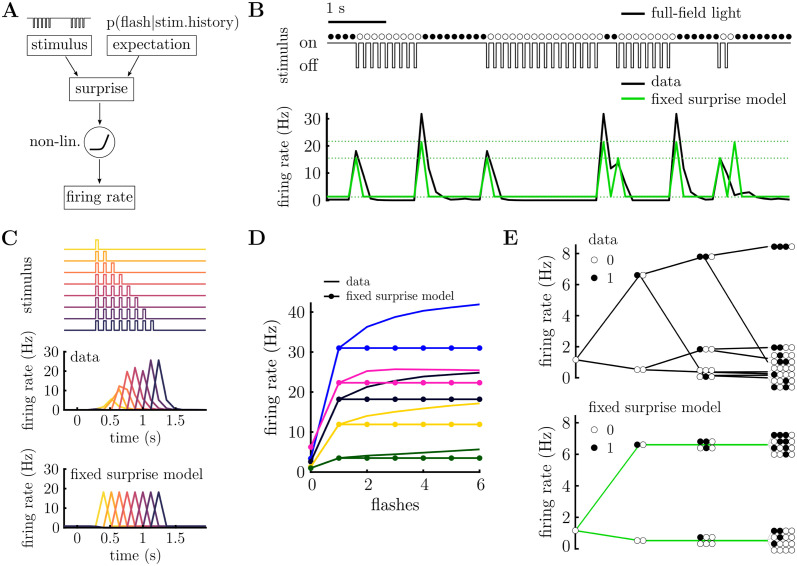
Fixed surprise model. **A.** Schematic of modeling framework. The stimulus is compared to the neuron’s expectation, which depends on their internal model, to compute surprise. The encoded surprise is then transformed via a static non-linearity to obtain the neuron’s firing rate. **B.** Stimulus excerpt (above) and recorded PSTH (below, black), and prediction of the fixed surprise model (below, green). The fixed surprise model has limited flexibility, only permitting four possible firing rates (indicated with dashed lines). **C.** Response to flash sequences of varying length (top). PSTH for a single neuron (middle) and model prediction (bottom) to the stimulus sequences shown above. Each colour corresponds to a different length of flash sequence. The fixed surprise model predicts the OSR magnitude to be independent of the number of flashes. **D.** OSR for 5 cells (solid lines) after a varying number of consecutive flashes. The fixed surprise model (lines with filled circles) cannot account for the increase in the OSR with increasing number of flashes. **E.** Tree-plot for a single cell (above) and fixed model prediction (below). The fixed surprise model can capture the mean response for stimulus sequences of up to length 2, but not beyond. Tree-plots corresponding to sequences ending with a flash are shown in S2 Fig.

We asked whether RGC responses were consistent with them encoding surprise. To test this, we constructed a simple model of how RGCs could combine their internal stimulus expectations with their recent stimulus history to compute surprise (Fig 2A). Following [18, 19], we defined surprise at time *t*, *s*_*t*_, as the negative log probability of a stimulus, *x*_*t*_, given the recent stimulus history, *x*_<*t*_, and the neuron’s internal model of the stimulus statistics (parameterised by *θ*):

$$\begin{array}{c} s_{t}=-\text{log}\;p(x_{t}|x_{<t},\theta ) \end{array}$$ (16)

The mean firing rate was then obtained by applying a simple non-linear mapping:

$$\begin{array}{c} r_{t}=f(as_{t}+b) \end{array}$$ (17)

where *a* and *b* are free parameters and *f*(⋅) was assumed to be a softplus (log(1 + *e*^*x*^)) non-linear function to prevent firing rates being negative.

The computed ‘surprise’ for each cell thus depends on their expectations or ‘internal model’ of the stimulus statistics (parameterized by *θ*). We first assumed the simplest possible internal model: a Markov model, in which the probability of observing a flash, *x*_*t*_ = 1, only depends on whether there was a flash or not in the previous time bin (*x*_*t*_ = 0/1). We called this the ‘fixed surprise’ model, since the surprise is fixed by the stimulus presented in the previous time-step. This fixed surprise model has two free parameters: the probability of a flash occurring if there was/wasn’t a flash in the previous time-step (*θ*_0_ = *p*(*x*_*t*_ = 1|*x*_*t*−1_ = 0), and *θ*_1_ = *p*(*x*_*t*_ = 1|*x*_*t*−1_ = 1)). The parameters of the response function (*a* and *b*) and internal model (*θ*) were fitted for each neuron using maximum likelihood, assuming that the responses were generated by a Poisson distribution with mean *r*_*t*_ (see Methods: Neural model).

Fig 2B shows the average firing rate of a single neuron (black) for a given stimulus sequence (above) (see Methods: Data analysis). This model accounted for the most prominent feature of the neuron’s responses: that it responded strongly to the first flash in a sequence, and the first silence in a sequence (i.e. the OSR). However, the model was unable to replicate the dependence of the OSR on the number of flashes presented in a row, observed for this (Fig 2C) and many other cells (Fig 2D). This was because, by design, with a Markov model the computed surprise, only depends on the stimulus in the previous time-bin, and thus only two responses to a silence are possible, depending on whether there was a flash or not in the previous time-step (Fig 2E; see S2 Fig for complementary tree-plot, for sequences ending with a flash).

### Adaptive surprise model

To account for the observed variations in the OSR with number of consecutive flashes, we next considered a more complex ‘dynamic belief’ internal model. Here, we assume that the transition probabilities (*θ*_*i*_ ≡ *p*(*x*_*t*_ = 1|*x*_*t*−1_ = *i*)), are not known *a priori* by each neuron, but must be inferred. We assume neurons combine their prior expectations (*p*(*θ*_*i*_)) with the recent stimulus history (*p*(*x*_*t*_, *x*_*t*−1_, …|*θ*)) using Bayes’ law: *p*(*θ*|*x*_*t*_, *x*_*t*−1_) ∝ *p*(*x*_*t*_, *x*_*t*−1_, …|*θ*_*i*_)*p*(*θ*_*i*_). We assumed a beta-distribution for the prior over *θ*_*i*_, with parameters *α*_*i*_ and *β*_*i*_ of the form: $p(\theta_{i})=\theta_{i}^{\alpha_{i}-1}{\left(1-\theta_{i}\right)}^{\beta_{i}-1}$. Multiplying the beta prior with the Bernoulli distribution, *p*(*x*_*t*_, *x*_*t*−1_, …|*θ*_*i*_), we obtain:

$$\begin{array}{c} p(\theta_{i}|x_{t},x_{t-1},\ldots )\propto p(x_{t},x_{t-1},\ldots |\theta_{i})p(\theta_{i})=\theta^{n_{i\to 1}+\alpha_{i}-1}{\left(1-\theta \right)}^{n_{i\to 0}+\beta_{i}-1} \end{array}$$ (18)

where *n*_*i*→*j*_ is the number of occurrences of the transition *i* → *j* in the sequence {*x*_1_, *x*_2_, …, *x*_*t*_}, and *α*_*i*_ and *β*_*i*_ are parameters of the prior. Finally, we integrate out *θ*, to obtain a simple expression for the inferred probability of observing *x*_*t*_ = 1, given *x*_*t*−1_ = *i*:

$$\begin{array}{c} p(x_{t}=1|x_{t-1}=i,x_{t-2},\ldots )={\left\langle \theta \right\rangle }_{p(\theta_{i}|x_{t},x_{t-1},\ldots )}=\frac{n_{i\to 1}+\alpha_{i}}{n_{i\to 0}+n_{i\to 1}+\beta_{i}+\alpha_{i}}, \end{array}$$ (19)

We assume that the parameters of the prior (*α*_*i*_, *β*_*i*_) are different for each neuron. Note that, in the limit where the prior is very strong (i.e. *n*_*i*→*j*_ ≪ *α*_*i*_ and *n*_*i*→*j*_ ≪ *β*_*i*_), this model becomes identical to the ‘fixed-belief’ model described previously (Fig 2), where the inferred transition probabilities do not depend on the stimulus history, beyond the previous time-step.

If neurons had ‘infinite’ memory then, given a sufficiently long stimulus sequence, their prior expectations would have no effect. Instead, we assume a more biologically plausible model where neuron’s have a finite memory, and *n*_*j*→*i*_ are estimated using a leaky integration of past observations (see Methods: Adaptive surprise model). This requires one additional parameter (the time-scale of integration i.e. the leak parameter), which we kept fixed for all neurons. In S1 Text we show how qualitatively similar results can be obtained by assuming neurons perform Bayesian inference, given a model where the transition probabilities have a small probability of changing on each time-step. However, optimal Bayesian inference in this setting required complex numerical integration, and it is thus hard to see how it could be implemented feasibly by individual neurons. As a result, we focus on the simpler ‘leaky integration’ model for the rest of the paper.

**Fig 3:**
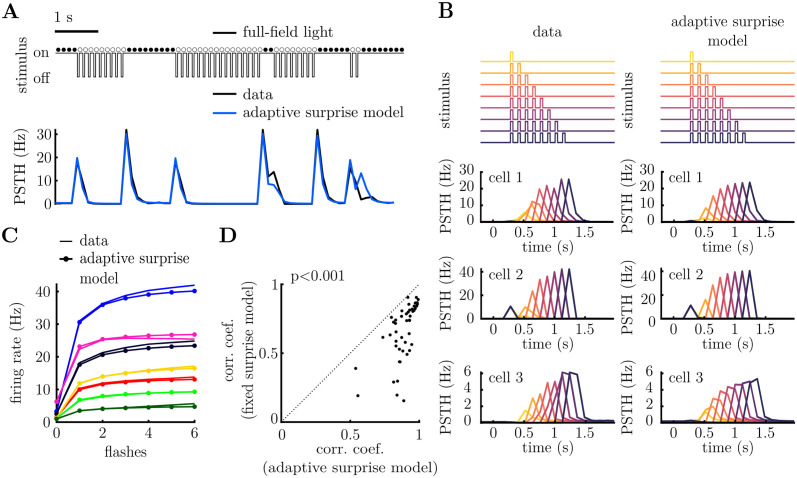
Adaptive surprise model. **A.** Stimulus excerpt (above) and recorded PSTH (below, black), alongside prediction of the adaptive surprise model (below, blue). **B.** Neural responses to varying number of consecutive flashes (above). Recorded PSTH of three neurons is shown to the left, while model predictions are shown to the right. Each colour corresponds to a different number of consecutive flashes. The adaptive surprise model captures variations in both the magnitude and width of the OSR. **C.** Increase in the OSR with the number of consecutive flashes for seven cells (each cell plotted with a different colour). The data (solid line) is plotted alongside the predictions of the adaptive surprise model (solid lines with circles). **D.** Pearson correlation coefficients between each cell’s PSTH and the model predictions, for the fixed surprise model (y-axis) versus the adaptive surprise model (x-axis). The adaptive surprise model significantly outperforms the fixed surprise model (p = 1 ⋅ 10^−9^, Wilcoxon signed-rank test).

We fitted the 4 parameters of the prior (plus the gain and bias of the LN model, corresponding to *a* and *b* in Eq 2, respectively) for each neuron, using maximum likelihood, assuming Poisson noise [20]. S12 Fig shows the fitted prior for 6 representative cells. Fig 3A shows the predicted firing rate for one neuron (blue) to a short stimulus sequence (above). The ‘adaptive surprise’ model was able to capture aspects of the neuron’s response that could not be accounted for by the previous ‘fixed surprise model’. For example, it could capture how the size of the OSR increased with the number of flashes presented in a row (Fig 3B and 3C). Further, it captured individual differences in the OSR decay for different neurons (compare cells 1–3 in Fig 3B). Overall, the correlation between the estimated firing rates and the model prediction was significantly higher for the adaptive, compared to the fixed, surprise model (Fig 3D).

**Fig 4:**
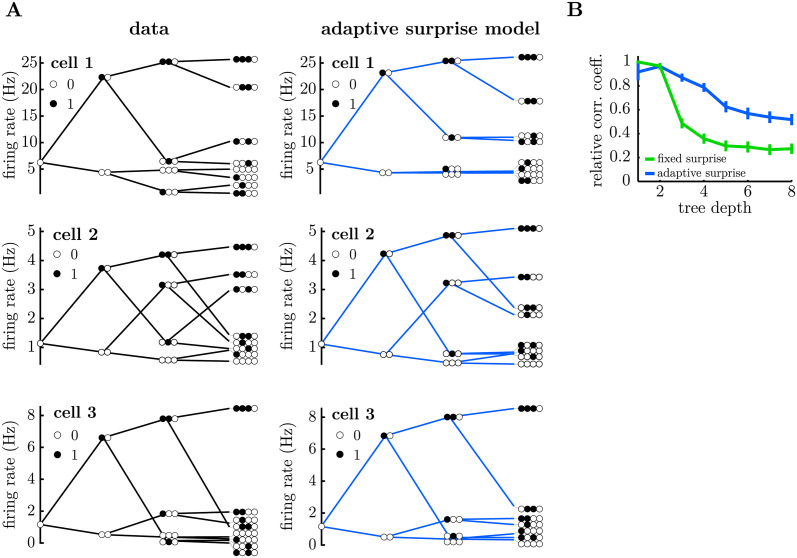
Neural responses to different stimulus sequences, for the adaptive surprise model. **A.** Tree-plot, showing the mean response of three representative cells to all possible sequences of flashes (filled circles) and silences (empty circles) of a given length. As we move rightward, the tree branches to show responses to take into account stimuli presented further in the past. The data is shown on the left and the model predictions on the right. The adaptive model is a able to reproduce qualitative aspects of each tree-plot, beyond the top branch (which shows how the OSR magnitude varies with the number of consecutive flashes). Tree-plots corresponding to sequences ending with a flash are shown in S3 Fig. **B.** Correlation coefficient between the tree-plot obtained with the adaptive surprise model and the data, computed separately for each tree-depth (i.e. stimulus sequence length). The adaptive model is significantly better at capturing the shape of the tree for stimulus sequences longer than 2, compared to the fixed surprise model.

To further investigate how the adaptive surprise model could account for the diverse responses of different cells, we plotted tree-plots showing the average firing rates predicted by the model for stimulus sequences of different lengths (Fig 4A; see S3 Fig for complementary tree-plot, for sequences ending with a flash). The adaptive belief model captured much of the structure in the neural responses to stimulus sequences of varying length, as well as the diversity across different cells. This was supported by plotting the correlation coefficient between the model predictions for each node of the tree and the data, which decayed slowly with the tree depth (Fig 4B), compared to the fixed surprise model which reduced dramatically for tree depth greater than 2. Finally, S10 Fig shows that our model can account for how neural responses depend on stimuli further in the past, quantified by how much different nodes branch out from their parent node as we increase the depth of the tree plot.

**Fig 5:**
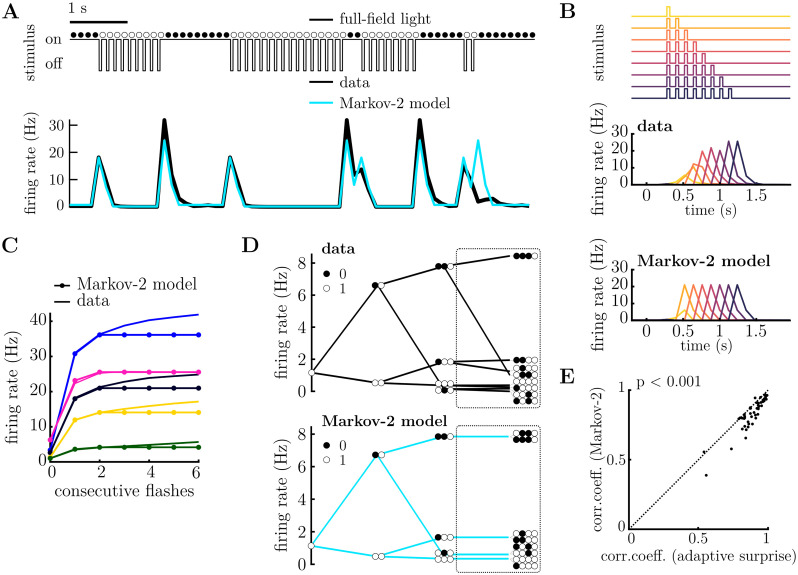
Fixed surprise model with longer past (Markov-2 model). **A.** Stimulus excerpt (above) and recorded PSTH (below, black), and firing rate predicted by the Markov-2 model (below, blue). **B.** Response to varying number of consecutive flashes (top). PSTH for a single neuron (middle) and model prediction (below) to the stimulus sequences shown above. Each colour corresponds to a different length of flash sequence. The Markov-2 surprise model predicts the OSR magnitude to be dependent on the previous two state only. **C.** Average responses of 5 cells (solid lines) to flash sequences of varying lengths. The Markov-2 surprise model (lines with filled circles) cannot account for the increase in the OSR beyond 2 consecutive flashes. **D.** Tree-plot for a single cell (above) and fixed model prediction (bottom). The Markov-2 surprise model cannot capture the response for stimulus sequences greater than length 2 (highlighted with dashed circle). Tree-plots corresponding to sequences ending with a flash are shown in S4 Fig. **E.** The correlation coefficient between the adaptive surprise model and recorded PSTH for each cell is significantly better than for the Markov-2 model (p = 2 ⋅ 10^−8^, Wilcoxon signed-rank test).

To further test our adaptive belief model, we compared it to a more complex fixed belief model, with a comparable number of free parameters. To do this, we implemented a ‘Markov-2 model’ in which the probability of observing a flash is depends on the observed stimulus in the previous two time-bins. This model’s prior has 4 parameters, (*θ*_*ij*_ = *p*(*x*_*t*+1_ = 1|*x*_*t*_ = *i*, *x*_*t*−1_ = *j*)), which is the same as the adaptive surprise model (aside from the leak parameter, which we kept the same for all cells). The behaviour of this model is shown in Fig 5. While the Markov-2 model outperformed the fixed surprise model model described earlier, it could not, by construction, account for increases in the OSR that occurred for sequences of more than 2 consecutive flashes (Fig 5B and 5C), or any structure in the tree plots at a depth greater than 2 (Fig 5D, emphasized with a dashed ellipse). Finally, the correlation coefficient between predicted and observed firing rates was significantly worse for the Markov-2 model than the adaptive surprise model (Fig 5E) despite them having the same number of free parameters (*p* = 2 ⋅ 10^−8^, Wilcoxon signed-rank test).

### Differences in the internal expectations for individual cells

**Fig 6:**
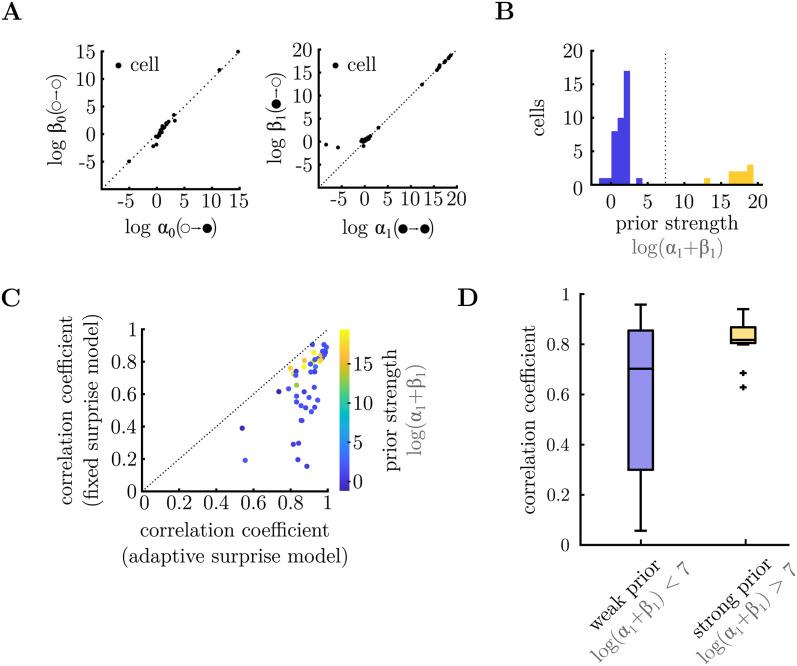
Parameters of internal model. **A.** Parameters of the inferred prior (*α*_*i*_ and *β*_*i*_) for each cell. These parameters determine each cell’s prior expectation for the different transitions from silence (left) or flash (right). In both cases, the ratio between these parameters, *α*_*i*_/*β*_*i*_ (which determine the mean of the prior) is close to unity for all the cells, while their sum, *α*_*i*_ + *β*_*i*_ (which determines the strength of the prior) varies across different cells. **B.** Histogram of log(*α*_1_ + *β*_1_) for different cells. The population could be split into two groups: cells with low confidence in the prior (i.e. small *α*_*i*_ + *β*_*i*_; blue) and cells with high confidence in the prior (i.e. large *α*_*i*_ + *β*_*i*_; yellow). **C.** Correlation coefficient between the responses predicted by the adaptive surprise model, versus the fixed surprise model. Each circle is colour coded according to the parameters of the inferred prior for that cell log(*α*_1_ + *β*_1_). Cells that had a strong prior (yellow) tended to be better fit by the fixed surprise model, relative to the adaptive surprise model. **D.** For each cell we computed the correlation coefficient between the average neural responses to stimulus sequences of length 2, versus average responses that take into account stimulus sequences of length 10. A correlation of 1 would imply that neural responses just depended on the stimulus in the previous time-step; values less than 1 imply that neural responses depend on stimuli further in the past. As expected, the responses of cells with a strong prior (right, yellow) exhibited a higher correlation coefficient than cells with a weak prior (left, blue), showing that they could be better predicted just by looking at the most recent stimulus transition.

We were interested to see how the inferred expectations (the ‘prior’) varied for each cell. Recall that we assumed a beta-prior over the transition probabilities *θ*_*i*_ = *p*(*x*_*t*_ = 1|*x*_*t*−1_ = *i*), with parameters *α*_*i*_ and *β*_*i*_. The mean of this prior is determined by the ratio of these two parameters, *α*_*i*_/*β*_*i*_, while its width (i.e. the level of prior uncertainty) is determined by their sum, *α*_*i*_ + *β*_*i*_. Fig 6A and 6B show how the parameters of the prior varied for different cells. Interestingly, we found that while for different cells there was a large variation in the sum, *α*_*i*_ + *β*_*i*_, the ratio, *α*_*i*_/*β*_*i*_, was relatively constant. Thus, while the width of the prior, which determines how much weight is accorded to prior expectations versus new observations, varied greatly across cells, the prior mean was roughly constant.

Focusing on the prior parameters, *α*_1_ and *β*_1_, which determines neural responses to ‘flash→flash’ and ‘flash→no-flash’ transitions (i.e. the OSR), we observed two clusters of cells (Fig 6A, right panel), with different levels of prior uncertainty (determined by the sum, *α*_1_ + *β*_1_; Fig 6B). We asked what effect this would have on these cells responses. We reasoned that cells with a strong prior (i.e. large *α*_*i*_ + *β*_*i*_) would not adapt their posterior belief much depending on recent observations, and hence their responses would be well predicted by the fixed surprise model. In contrast, cells with a weak prior (i.e. small *α*_*i*_ + *β*_*i*_) would be strongly influenced by recent observations, and thus their responses would be poorly predicted by the fixed-surprise model. This turned out to be the case. Fig 6C shows the correlation coefficient between the prediction of the fixed surprise model and recorded responses, versus the adaptive surprise model. There was a trend for cells with a strong prior (high *α*_*i*_ + *β*_*i*_; colour coded in yellow) to be equally well-fit by both models, while cells with a weak prior (low *α*_*i*_ + *β*_*i*_; colour coded in blue) were better fit by the adaptive surprise model.

We next asked whether this effect could be observed in the data, without reference to the model fits. To do this, we compared the average neural responses to stimulus sequences of length 2, to average responses to longer stimulus sequences, of length 10. A correlation of 1 would imply that neural responses just depended on the stimulus in the previous time-step; values less than 1 imply that neural responses depend on stimuli further in the past. As expected, we found that cells with a strong prior (i.e. log (*α*_1_ + *β*_1_) > 7) exhibited high correlation coefficients, close to 1, implying that their responses could be well predicted by just looking at the most recently presented stimuli (Fig 6D, yellow). This was not the case for stimuli with a weak prior (Fig 6D, blue) (p = 0.066, Wilcoxon rank-sum test).

**Fig 7:**
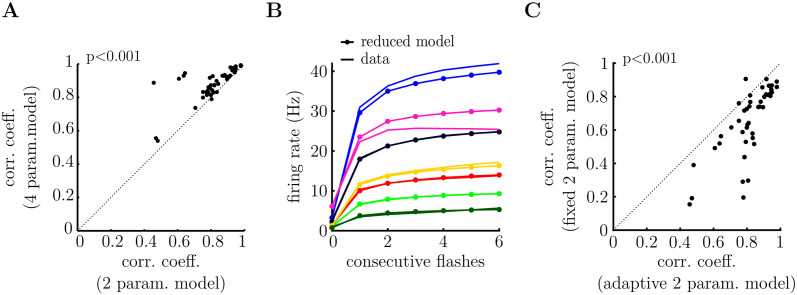
Reduced adaptive surprise model, with a fixed prior mean (i.e. *α*_*i*_/*β*_*i*_ = 1). **A.** The reduced model performs almost as well as the adaptive surprise model despite having half the number of free parameters. (p = 3 ⋅ 10^−9^, Wilcoxon signed-rank test). **B.** Mean response of 7 cells following a variable number of flashes presented in a row. The increase in the OSR with the number of flashes is well-fitted by the reduced model. **C.** The reduced adaptive surprise model performs significantly better at fitting the recorded neural responses, despite both models having the same number of free parameters per cell. (p = 4 ⋅ 10^−5^, Wilcoxon signed-rank test.).

In Fig 6A we observed that the prior mean, determined by *α*_*i*_/*β*_*i*_, remained near-unity across different cells. We thus, asked whether it would be possible to fit neural responses using a reduced adaptive surprise model with only two parameters (i.e. the sum, *α*_*i*_ + *β*_*i*_), and *α*_*i*_/*β*_*i*_ held fixed at unity. Fig 7A shows that, while this reduced adaptive model performed worse than the full adaptive surprise model, this reduction in performance was small (< 10% reduction in correlation coefficient), despite having having only 2 free parameters per cell (compared to 4 parameters, for the full model). Notably, the reduced model was able to capture similar qualitative features of neural responses, such as how the OSR increased with the number of consecutive flashes (Fig 7B and S5 Fig). Its performance was also significantly better than the fixed surprise model, which had the same number of free parameters per cell (p = 4 ⋅ 10^−5^, Wilcoxon signed-rank test). Tree-plots for the reduced adaptive surprise model are shown in S4 Fig.

## Discussion

We observed how neural responses in the retina showed non-trivial dependencies on the precise order of flashes and silences in random stimulus sequences (Fig 1C and 1D). Interestingly, RGC responses were well predicted by a simple model, which assumed that they depended on how ‘surprising’ stimuli were, relative to an internally generated expectation (Figs 3 and 4). Moreover, our model showed how the different ‘expectations’ of different neurons could account for the diverse way they responded to presented stimuli (Figs 6 and 7).

Our approach contrasts with previous ideal observer models, which assume that neurons are perfectly adapted to the ‘true’ presented stimulus statistics [21–23]. Instead, we found that neural responses could be well explained by assuming that each neuron has learned its own internal model of the stimulus statistics (with the parameters of the prior fitted separately for each cell). Interestingly, we found that different neurons had very similar prior expectations about which stimuli were most likely to occur (determined by the mean of the prior). What varied was the degree of confidence they had about their own prior expectations (determined by the width of the prior). Furthermore, recorded cells could be divided into two categories: those with weak confidence in their prior, and those with strong confidence in their prior expectations. We were interested to see whether this split was related to different functional cell types. To do this, we performed a second experiment, where we presented a full-field ‘chirp’ stimulus, which has been used previously to classify different cell-types in the mouse [24]. We confirmed that the main results with this second experiment were qualitatively identical (S6 Fig). We then performed clustering based on the responses of different cells to the chirp stimulus (S7 Fig). We did indeed find a trend for some cell types to exhibit an OSR (S8 Fig), as well as for some types to show larger/stronger fitted priors (S9 Fig). However, the fitted prior would be clearly insufficient to identify the cell type alone, since there was a large spread of parameters within each cell type. Further experiments would be required to investigate further the relation between surprise encoding and cell type.

Our modelling framework was adapted from a previous model of Meyniel et al., that sought to explain psychophysical data showing how subjects’ behaviour (such as their reaction time and accuracy) depended on the statistics of sequentially presented sensory stimuli [16]. Meyniel and colleagues showed how their data could by explained if subjects used a Bayesian inference model, as described here, to predict new stimuli based on what came before. Here, we extended this model to include a variable ‘prior’ distribution, whose parameters could be fit to describe the diverse responses of different ganglion cells. Nonetheless, the fact that a similar type of model can be used to describe both neural responses in the retina and subjects behaviour in different tasks is intriguing, raising the question of whether similar computations may be present ubiquitously in the brain when subjects are presented stimuli with complex temporal statistics. This is an important link to explore, since many previous experiments have shown how our perceptual experience is flexible, and can be adapt depending on the recent stimulus history [25, 26].

Previously it was shown that in certain cases, retinal neurons are able to adapt to the statistics of their environment, so as to preferentially encode stimuli that are most unpredictable in any given context [27, 28]. Recently, Mlynarski & Hermundstadt developed a mathematical framework to account for how such adaptive efficient coding could be performed by sensory neurons [29, 30]. The question remains however, over which timescales, and which type of stimulus statistics, retinal neurons are able to adapt to. In our experiment, we found that RGCs adapt to recent stimulus statistics: notably the sequence of flashes presented over a period of 1–2 seconds. While beyond the scope of the current work, previous studies have proposed several possible mechanisms for this, including depressing inhibitory synapses [31] and combined ON and OFF pathways [13] (S11 Fig). On the other hand, however, we found little evidence that RGCs could adapt to stimulus statistics over longer time-scales. This was evidenced by the fact their learned prior differed significantly from the stimulus statistics presented over the course of the experiment. Further, we found that introducing subtle changes to the stimulus statistics (e.g. increasing the probability of either very long or short flash sequences, while keeping the average number of flashes presented in a row the same), had little effect on the resulting RGC response properties in our experiment. It would be interesting investigate this further in the future to see, for example, how and whether RGC responses vary depending on more strong changes in the stimulus statistics, including in the presence of more complex, naturalistic, stimuli.

Previous experimental [12, 13, 32] and computational [33–35] studies sought to understand the neural mechanisms underlying the OSR. However, there remains some controversy over which of the proposed theories could explain all of the experimentally observed features of the OSR, such as e.g. the fact that the delay before the OSR varies linearly with the time between flashes. Our work provides further constraints to distinguish between different theories, by showing how the OSR varies depending on the precise sequence of flashes and silences (Fig 1).

The stimuli in our experiment, which consisted of sequences of full-field flashes, were chosen to be sufficiently rich so as to permit many different levels of ‘surprise’, while simple enough to permit a straight-forward analysis of neural responses. Nonetheless, in the future it would be interesting to investigate neural responses to more naturalistic stimuli, which for example, varied spatially as well temporally [36]. This would allow us to investigate, for example, the degree to which neurons’ internal model is adapted to the statistics of natural scenes, as predicted by the efficient coding hypothesis [37].

## Supporting information

S1 Fig Tree-plot for two representative cells, corresponding to sequences ending with a flash. Here we show the mean response of two representative cells to different sequences of flashes (filled circles) and silences (empty circles). Each column of the tree-plot shows the average response of the neuron to all stimulus sequences of a given length, that end with flash.
(PDF)

S2 Fig Tree-plot for a single cell (left) and fixed surprise model prediction (right). Each column of the tree-plot shows the average response of the neuron to all stimulus sequences of a given length, that end with flash.
(PDF)

S3 Fig Tree-plot for three representative cells, showing neuron’s response (left column), and prediction by the adaptive surprise model (right column). (PDF)

S4 Fig Tree-plot for reduced adaptive surprise model, showing neuron’s response (left column), and prediction by the adaptive surprise model (right column). The qualitative traits of the adaptive surprise model remain even if two instead of four prior parameters are used.
(PDF)

S5 Fig Dynamic inference model performs similar to its leaky integration approximation. **A.** Stimulus excerpt (above) and recorded PSTH (below, black), and prediction of the dynamic surprise model (below, red). **B.** Pearson correlation coefficients of model fits to each cell’s PSTH, for the dynamic surprise model (x-axis) versus the adaptive surprise model (y-axis). Two model perform similarly (the correlation coefficients for the respective model fits lie close to the unity line) despite the significant difference in mean (p = 3 ⋅ 10^−5^, Wilcoxon signed-rank test). As such, the adaptive surprise model can be treated as an approximation of the dynamic surprise model. **C.** Tree-plot, showing the mean response of two representative cells to different sequences of flashes (filled circles) and silences (empty circles). Each column of the tree-plot shows the average response of the neuron to all stimulus sequences of a given length, that end with silence (top) or flash (bottom).
(PDF)

S6 Fig Adaptive surprise model for a repeated experiment. **A.** Stimulus excerpt (above) and recorded PSTH (below, black), alongside prediction of the adaptive surprise model (below, blue). **B.** Neural responses to varying number of consecutive flashes (above). Recorded PSTH of three neurons is shown to the left, while model predictions are shown to the right. Each colour corresponds to a different number of consecutive flashes. The adaptive surprise model captures variations in both the magnitude and width of the OSR. **C.** Increase in the OSR with the number of consecutive flashes for seven cells (each cell plotted with a different colour). The data (solid line) is plotted alongside the predictions of the adaptive surprise model (solid lines with circles). **D.** Pearson correlation coefficients between each cell’s PSTH and the model predictions, for the fixed surprise model (y-axis) versus the adaptive surprise model (x-axis). The adaptive surprise model significantly outperforms the fixed surprise model (p = 1 ⋅ 7^−20^, Wilcoxon signed-rank test).
(PDF)

S7 Fig Cell typing for repeated experiment. (Top left panel) Full-field ‘chirp’ stimulus used to categorise different cell-types in the repeat experiment. (Lower left panels) Mean (red) and standard deviation (gray) of PSTH in response to the chirp stimulus after clustering into 8 different cell-types. (Right panels) Average temporal STA in response to binary checkerboard stimulus. Each categorised cell-type was further classified manually into putative ON, OFF & ON-OFF types, depending on their response to a prolonged on and off flash, at the start of the chirp stimulus.
(PDF)

S8 Fig Putative cell types in the second experiment, based on responses to chirp stimulus. For three identified cell types (ON, OFF, ON-OFF), we show the number of cells with/without an OSR.
(PDF)

S9 Fig Distribution of the fitted prior depending on the cell type. Each dot represents a cell. Cells have been clustered as in S7 Fig. We have also indicated how each of the broad groups divides into the ON, OFF, ON-OFF categories.
(PDF)

S10 Fig Tree history: Individual examples and population average. **A.** We wanted to quantify how neural responses depended on stimuli going back in the past. To do this, we quantified the ‘branching distance’ at each depth of the tree-plot, defined as the difference between branches that extending from the same ‘parent’ node. Plotted here are the results for the three cells shown in Fig 4. The adaptive surprise model was able to capture the qualitative shape of this curve for these cells. **B.** Population average of the branching distance (bars represent standard error). The main difference between data and model was that the branching distance decreases to zero for the model as the tree depth increased, unlike the data. This is likely due to the noise in the empirical estimates of firing rate from data, which results in small random variations in the positions of the branches in the tree plot.
(PDF)

S11 Fig **A.** Response of a model cell, using the model of Werner et al. [13]. **B.** Omitted stimulus response versus number of consecutive flashes, for one cell predicted by the Werner model. **C.** When we fitted the 3 free parameters of the model to our data, it was not able to fit the responses as well as the adaptive surprise model, with signicantly lower correlation coefficient (p = 2 ⋅ 10^−9^, Wilcoxon signed-rank test).
(PDF)

S12 Fig Learned priors over the transition probability, *θ* = *p*(*x*_*t*_ = 1|*x*_*t*−1_), fitted to data. Here we plot a subset of 6 cells.
(PDF)

S1 Text Dynamic surprise model. (PDF)

S2 Text Cell typing. (PDF)
