# Supplementary material for: Encoding surprise by retinal ganglion cells
Source: PLoS Comput Biol. 2024 Apr 17;20(4):e1011965. doi: 10.1371/journal.pcbi.1011965 (PMC11057717; doi:10.1371/journal.pcbi.1011965)
Supplement: S1 Fig — Here we show the mean response of two representative cells to different sequences of flashes (filled circles) and silences (empty circles). Each column of the tree-plot shows the average response of the neuron to all stimulus sequences of a given length, that end with flash. (PDF) [file pcbi.1011965.s001.pdf]

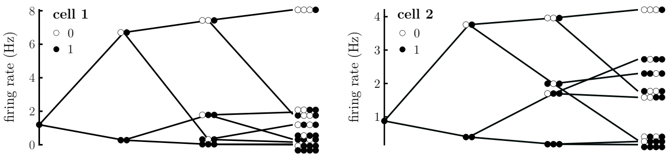

S1 Fig: Tree-plot for two representative cells in Fig 1D, corresponding to sequences ending with a flash. Here we show the mean response of two representative cells to different sequences of flashes (filled circles) and silences (empty circles). Each column of the tree-plot shows the average response of the neuron to all stimulus sequences of a given length, that end with flash.
