# Supplementary material for: Encoding surprise by retinal ganglion cells
Source: PLoS Comput Biol. 2024 Apr 17;20(4):e1011965. doi: 10.1371/journal.pcbi.1011965 (PMC11057717; doi:10.1371/journal.pcbi.1011965)
Supplement: S2 Fig — Each column of the tree-plot shows the average response of the neuron to all stimulus sequences of a given length, that end with flash. (PDF) [file pcbi.1011965.s002.pdf]

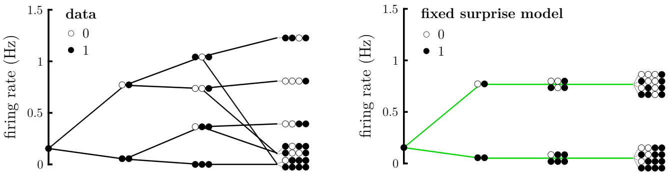

S2 Fig: Tree-plot for a single cell (left) and fixed surprise model prediction (right), complementary to tree shown in Fig 2E. Each column of the tree-plot shows the average response of the neuron to all stimulus sequences of a given length, that end with flash.
