# Supplementary material for: Encoding surprise by retinal ganglion cells
Source: PLoS Comput Biol. 2024 Apr 17;20(4):e1011965. doi: 10.1371/journal.pcbi.1011965 (PMC11057717; doi:10.1371/journal.pcbi.1011965)
Supplement: S3 Fig — (PDF) [file pcbi.1011965.s003.pdf]

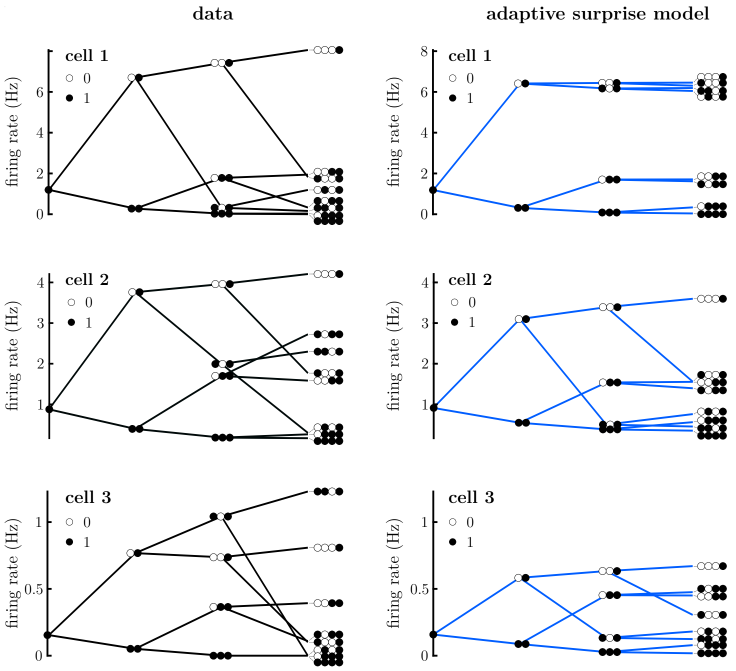

S3 Fig: Tree-plot for three representative cells featured in Fig 4A, showing neuron's response (left column), and prediction by the adaptive surprise model (right column).
