# Supplementary material for: Encoding surprise by retinal ganglion cells
Source: PLoS Comput Biol. 2024 Apr 17;20(4):e1011965. doi: 10.1371/journal.pcbi.1011965 (PMC11057717; doi:10.1371/journal.pcbi.1011965)
Supplement: S4 Fig — The qualitative traits of the adaptive surprise model remain even if two instead of four prior parameters are used. (PDF) [file pcbi.1011965.s004.pdf]

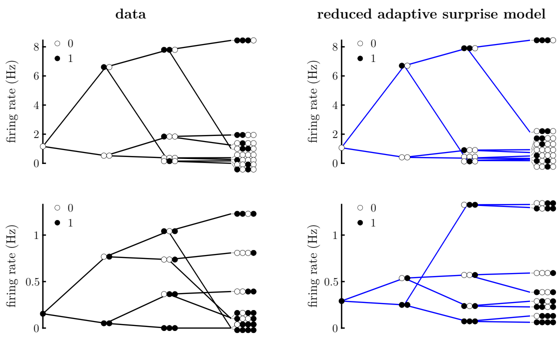

S4 Fig: Tree-plot for reduced adaptive surprise model, showing neuron's response (left column), and prediction by the adaptive surprise model (right column). The qualitative traits of the adaptive surprise model remain even if two instead of four prior parameters are used.
