# Supplementary material for: Encoding surprise by retinal ganglion cells
Source: PLoS Comput Biol. 2024 Apr 17;20(4):e1011965. doi: 10.1371/journal.pcbi.1011965 (PMC11057717; doi:10.1371/journal.pcbi.1011965)
Supplement: S5 Fig — A. Stimulus excerpt (above) and recorded PSTH (below, black), and prediction of the dynamic surprise model (below, red). B. Pearson correlation coefficients of model fits to each cell’s PSTH, for the dynamic surprise model (x-axis) versus the adaptive surprise model (y-axis). Two model perform similarly (the correlation coefficients for the respective model fits lie close to the unity line) despite the significant difference in mean (p = 3 ⋅ 10−5, Wilcoxon signed-rank test). As such, the adaptive surprise model can be treated as an approximation of the dynamic surprise model. C. Tree-plot, showing the mean response of two representative cells to different sequences of flashes (filled circles) and silences (empty circles). Each column of the tree-plot shows the average response of the neuron to all stimulus sequences of a given length, that end with silence (top) or flash (bottom). (PDF) [file pcbi.1011965.s005.pdf]

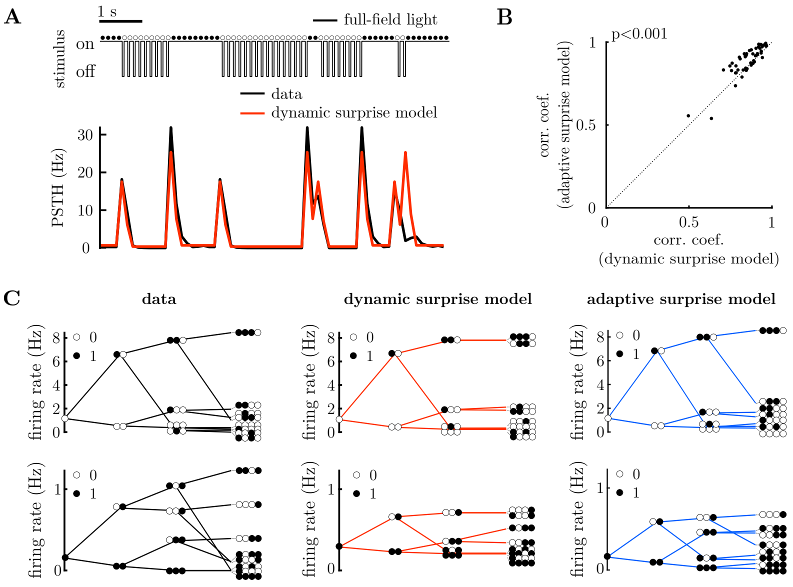

S5 Fig: **Dynamic inference model performs similar to its leaky integration approximation.** **A.** Stimulus excerpt (above) and recorded PSTH (below, black), and prediction of the dynamic surprise model (below, red). **B.** Pearson correlation coefficients of model fits to each cell's PSTH, for the dynamic surprise model (x-axis) versus the adaptive surprise model (y-axis). Two model perform similarly (the correlation coefficients for the respective model fits lie close to the unity line) despite the significant difference in mean ($p = 3 \cdot 10^{-5}$, Wilcoxon signed-rank test). As such, the adaptive surprise model can be treated as an approximation of the dynamic surprise model. **C.** Tree-plot, showing the mean response of two representative cells to different sequences of flashes (filled circles) and silences (empty circles). Each column of the tree-plot shows the average response of the neuron to all stimulus sequences of a given length, that end with silence (top) or flash (bottom).
