# Supplementary material for: Encoding surprise by retinal ganglion cells
Source: PLoS Comput Biol. 2024 Apr 17;20(4):e1011965. doi: 10.1371/journal.pcbi.1011965 (PMC11057717; doi:10.1371/journal.pcbi.1011965)
Supplement: S6 Fig — A. Stimulus excerpt (above) and recorded PSTH (below, black), alongside prediction of the adaptive surprise model (below, blue). B. Neural responses to varying number of consecutive flashes (above). Recorded PSTH of three neurons is shown to the left, while model predictions are shown to the right. Each colour corresponds to a different number of consecutive flashes. The adaptive surprise model captures variations in both the magnitude and width of the OSR. C. Increase in the OSR with the number of consecutive flashes for seven cells (each cell plotted with a different colour). The data (solid line) is plotted alongside the predictions of the adaptive surprise model (solid lines with circles). D. Pearson correlation coefficients between each cell’s PSTH and the model predictions, for the fixed surprise model (y-axis) versus the adaptive surprise model (x-axis). The adaptive surprise model significantly outperforms the fixed surprise model (p = 1 ⋅ 7−20, Wilcoxon signed-rank test). (PDF) [file pcbi.1011965.s006.pdf]

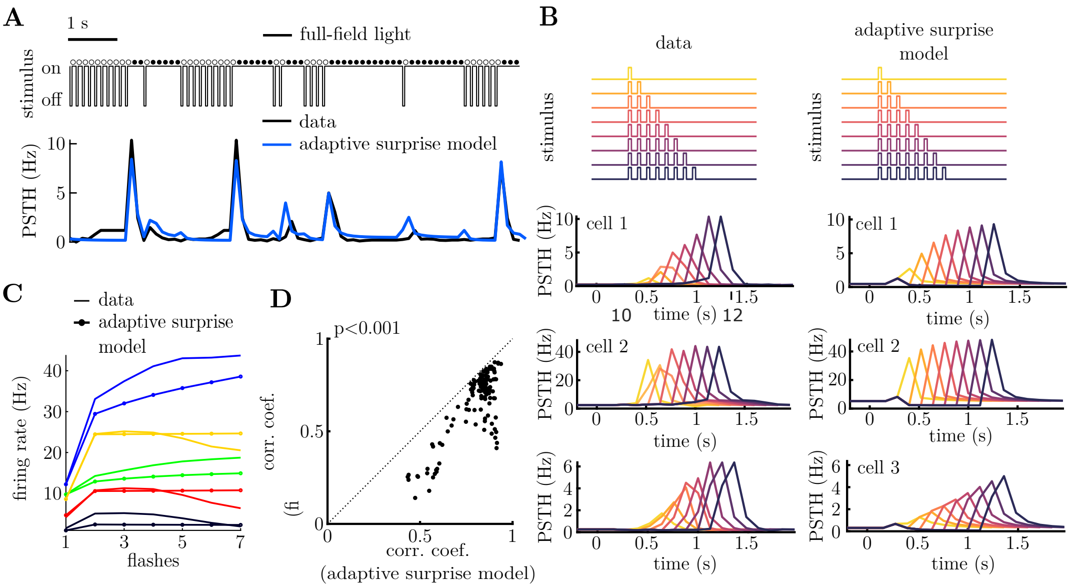

S6 Fig: **Adaptive surprise model for a repeated experiment.** **A.** Stimulus excerpt (above) and recorded PSTH (below, black), alongside prediction of the adaptive surprise model (below, blue). **B.** Neural responses to varying number of consecutive flashes (above). Recorded PSTH of three neurons is shown to the left, while model predictions are shown to the right. Each colour corresponds to a different number of consecutive flashes. The adaptive surprise model captures variations in both the magnitude and width of the OSR. **C.** Increase in the OSR with the number of consecutive flashes for seven cells (each cell plotted with a different colour). The data (solid line) is plotted alongside the predictions of the adaptive surprise model (solid lines with circles). **D.** Pearson correlation coefficients between each cell's PSTH and the model predictions, for the fixed surprise model (y-axis) versus the adaptive surprise model (x-axis). The adaptive surprise model significantly outperforms the fixed surprise model ($p = 1 \cdot 7^{-20}$, Wilcoxon signed-rank test).
