# Supplementary material for: Encoding surprise by retinal ganglion cells
Source: PLoS Comput Biol. 2024 Apr 17;20(4):e1011965. doi: 10.1371/journal.pcbi.1011965 (PMC11057717; doi:10.1371/journal.pcbi.1011965)
Supplement: S7 Fig — (Top left panel) Full-field ‘chirp’ stimulus used to categorise different cell-types in the repeat experiment. (Lower left panels) Mean (red) and standard deviation (gray) of PSTH in response to the chirp stimulus after clustering into 8 different cell-types. (Right panels) Average temporal STA in response to binary checkerboard stimulus. Each categorised cell-type was further classified manually into putative ON, OFF & ON-OFF types, depending on their response to a prolonged on and off flash, at the start of the chirp stimulus. (PDF) [file pcbi.1011965.s007.pdf]

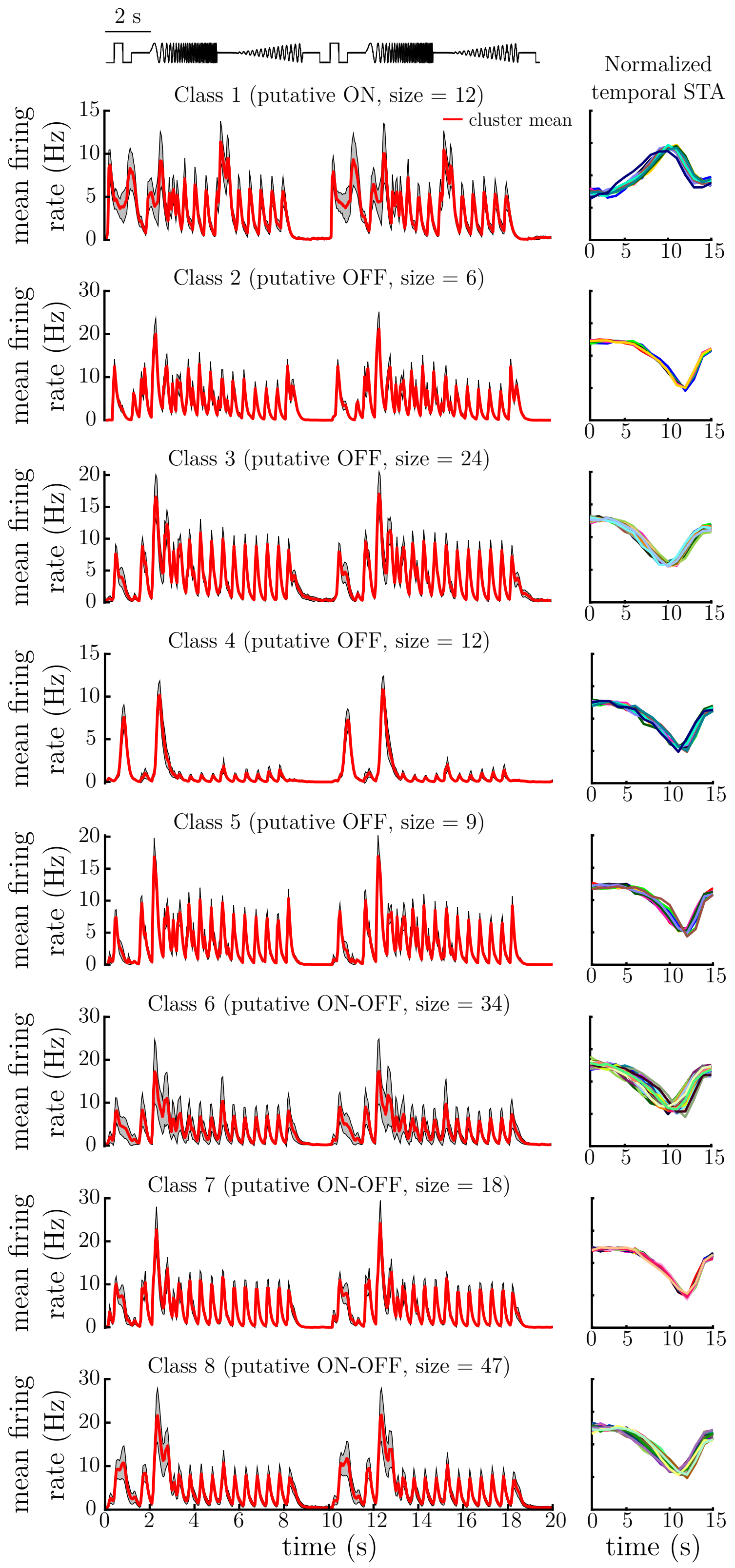

S7 Fig: **Cell typing for the repeated experiment.** (Top left panel) Full-field 'chirp' stimulus used to categorise different cell-types in the repeat experiment. (Lower left panels) Mean (red) and standard deviation (gray) of PSTH in response to the chirp stimulus after clustering into 8 different cell-types. (Right panels) Average temporal STA in response to binary checkerboard stimulus. Each categorised cell-type was further classified manually into putative ON, OFF ON-OFF types, depending on their response to a prolonged on and off flash, at the start of the chirp stimulus.
