# Supplementary material for: Encoding surprise by retinal ganglion cells
Source: PLoS Comput Biol. 2024 Apr 17;20(4):e1011965. doi: 10.1371/journal.pcbi.1011965 (PMC11057717; doi:10.1371/journal.pcbi.1011965)
Supplement: S8 Fig — For three identified cell types (ON, OFF, ON-OFF), we show the number of cells with/without an OSR. (PDF) [file pcbi.1011965.s008.pdf]

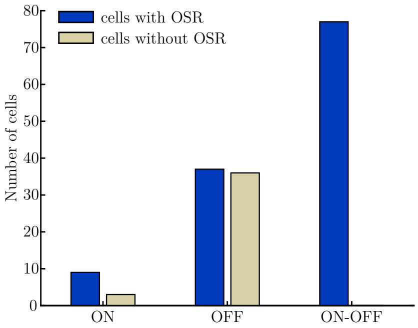

S8 Fig: **Putative cell types in the repeated experiment, based on responses to chirp stimulus.** For three identified cell types (ON, OFF, ON-OFF), we show the number of cells with/without an OSR.
