# Supplementary material for: Encoding surprise by retinal ganglion cells
Source: PLoS Comput Biol. 2024 Apr 17;20(4):e1011965. doi: 10.1371/journal.pcbi.1011965 (PMC11057717; doi:10.1371/journal.pcbi.1011965)
Supplement: S9 Fig — Each dot represents a cell. Cells have been clustered as in S7 Fig. We have also indicated how each of the broad groups divides into the ON, OFF, ON-OFF categories. (PDF) [file pcbi.1011965.s009.pdf]

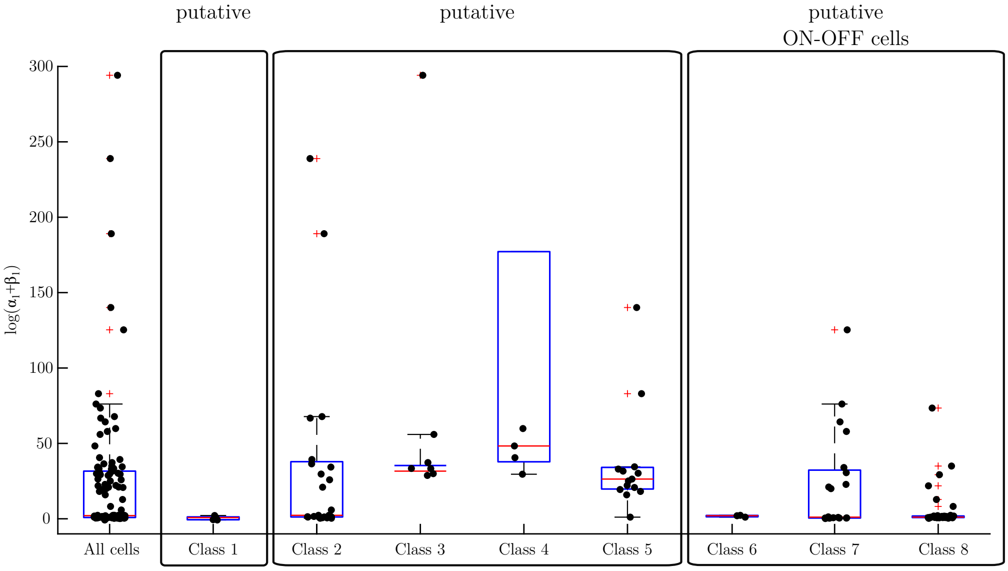

S9 Fig: **Distribution of the fitted prior depending on the cell type.** Each dot represents a cell. Cells have been clustered as in SI Fig S7. We have also indicated how each of the broad groups divides into the ON, OFF, ON-OFF categories.
