# Supplementary material for: Encoding surprise by retinal ganglion cells
Source: PLoS Comput Biol. 2024 Apr 17;20(4):e1011965. doi: 10.1371/journal.pcbi.1011965 (PMC11057717; doi:10.1371/journal.pcbi.1011965)
Supplement: S10 Fig — A. We wanted to quantify how neural responses depended on stimuli going back in the past. To do this, we quantified the ‘branching distance’ at each depth of the tree-plot, defined as the difference between branches that extending from the same ‘parent’ node. Plotted here are the results for the three cells shown in Fig 4. The adaptive surprise model was able to capture the qualitative shape of this curve for these cells. B. Population average of the branching distance (bars represent standard error). The main difference between data and model was that the branching distance decreases to zero for the model as the tree depth increased, unlike the data. This is likely due to the noise in the empirical estimates of firing rate from data, which results in small random variations in the positions of the branches in the tree plot. (PDF) [file pcbi.1011965.s010.pdf]

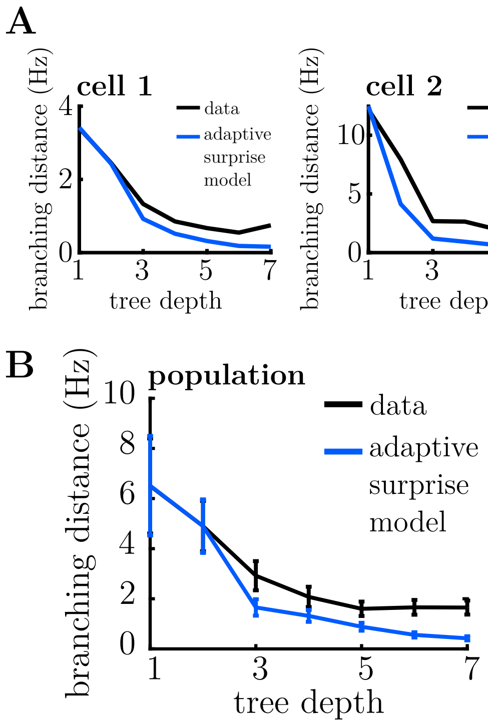

S10 Fig: **Tree history: individual examples and population average. A.** We wanted to quantify how neural responses depended on stimuli going back in the past. To do this, we quantified the 'branching distance' at each depth of the tree-plot, defined as the difference between branches that extending from the same 'parent' node. Plotted here are the results for the three cells shown in Figure 4. The adaptive surprise model was able to capture the qualitative shape of this curve for these cells. **B.** Population average of the branching distance (bars represent standard error). The main difference between data and model was that the branching distance decreases to zero for the model as the tree depth increased, unlike the data. This is likely due to the noise in the empirical estimates of firing rate from data, which results in small random variations in the positions of the branches in the tree plot.
