# Supplementary material for: Encoding surprise by retinal ganglion cells
Source: PLoS Comput Biol. 2024 Apr 17;20(4):e1011965. doi: 10.1371/journal.pcbi.1011965 (PMC11057717; doi:10.1371/journal.pcbi.1011965)
Supplement: S11 Fig — A. Response of a model cell, using the model of Werner et al. [13]. B. Omitted stimulus response versus number of consecutive flashes, for one cell predicted by the Werner model. C. When we fitted the 3 free parameters of the model to our data, it was not able to fit the responses as well as the adaptive surprise model, with signicantly lower correlation coefficient (p = 2 ⋅ 10−9, Wilcoxon signed-rank test). (PDF) [file pcbi.1011965.s011.pdf]

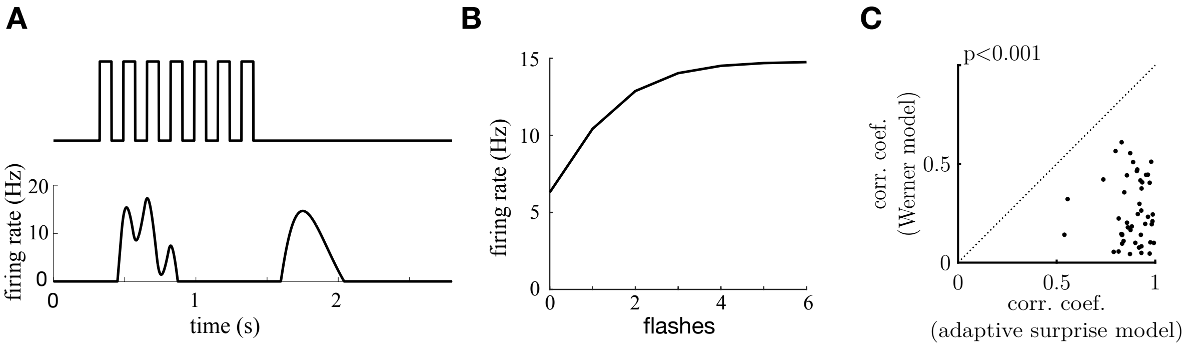

S11 Fig: Werner model applied to our experiment. **A.** Response of a model cell, using the model of Werner et al. [13]. **B.** Omitted stimulus response versus number of consecutive flashes, for one cell predicted by the Werner model. **C.** When we fitted the 3 free parameters of the model to our data, it was not able to fit the responses as well as the adaptive surprise model, with significantly lower correlation coefficient ($p = 2 \cdot 10^{-9}$, Wilcoxon signed-rank test).
