# Supplementary material for: Encoding surprise by retinal ganglion cells
Source: PLoS Comput Biol. 2024 Apr 17;20(4):e1011965. doi: 10.1371/journal.pcbi.1011965 (PMC11057717; doi:10.1371/journal.pcbi.1011965)
Supplement: S12 Fig — Here we plot a subset of 6 cells. (PDF) [file pcbi.1011965.s012.pdf]

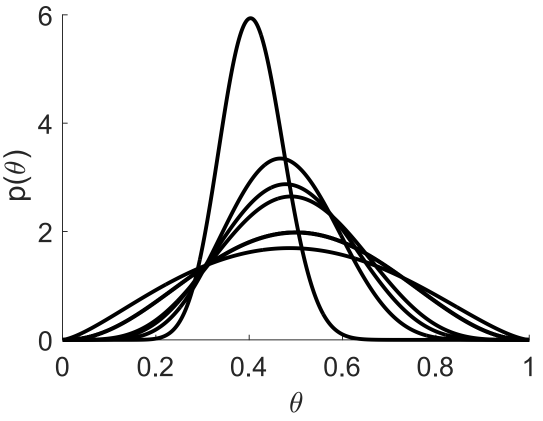

S12 Fig: Learned priors over the transition probability, $\theta = p(x_t = 1|x_{t-1})$, fitted to data. Here we plot a subset of 6 cells.
