# Supplementary material for: Encoding surprise by retinal ganglion cells
Source: PLoS Comput Biol. 2024 Apr 17;20(4):e1011965. doi: 10.1371/journal.pcbi.1011965 (PMC11057717; doi:10.1371/journal.pcbi.1011965)
Supplement: S1 Text — (PDF) [file pcbi.1011965.s013.pdf]

S1 Text: Dynamic surprise model

For the dynamic surprise model, the assumption is that the transition probability of a sequence can change at each time-step with a certain non-zero probability, p_c. However, as discussed in the main text, this model is both biologically implausible and computationally expensive, therefore the leaky integration model was used as its approximation. First, the probability p_c was learned independently for each cell. To better compare the performance of dynamic model with the adaptive model with fixed delay, the value of probability p_c was set to constant for all the cells and set at median value ($p_c = 0.2$). This value was the median of p_c. The model was fitted using algorithms with multiple starting points (MultiStart in MATLAB, 50 starting points, random initial parameters).
