# Supplementary material for: Encoding surprise by retinal ganglion cells
Source: PLoS Comput Biol. 2024 Apr 17;20(4):e1011965. doi: 10.1371/journal.pcbi.1011965 (PMC11057717; doi:10.1371/journal.pcbi.1011965)
Supplement: S2 Text — (PDF) [file pcbi.1011965.s014.pdf]

S2 Text: Cell typing

The temporal spike-triggered average (STA) was obtained using white noise checkerboard stimulus. The stimulus was a flickering black-and-white checkerboard where the intensity of each checker was randomly drawn from a binary distribution at every stimulus frame. The size of the checks was 60 μm, displayed at a frame rate of 30 Hz. The checkerboard stimulus was displayed in both discussed experiments. (Top left panel) In the repeated experiment we also displayed the full-field chirp stimulus [1]. This stimulus consists of two parts: in the first part, a one second flash of white light is followed by one second of dim background light; in the second part, there are two ramps lasting eight seconds each: during the first ramp, the luminance oscillates from dark to bright with constant contrast and increasing frequency; during the second ramp, the luminance oscillates with constant frequency and increasing contrast. This stimulus is shown in the top panel. The total duration of the chirp stimulus was 14 minutes, at a frame rate of 50 Hz.

The cell classification was performed using clustering on responses to the chirp stimulus, following method described in [2]. The minimal allowed cluster size was 5 cells, while the rest of the parameters were kept the same. Cells which did not fulfil the conditions of the clustering method (to have a good fit of the Gaussian distribution) were excluded, giving a total of 162 cells for which the type was determined. Out of those, 123 showed a clear OSR response.

The typing results for the second experiment (for which the chirp stimulus was presented) are shown in S7 Fig. Each row shows the mean response of the cell type to the chirp (left panels) and temporal STA (right). It is important to note that cell typing in salamander retina is not standardized, despite efforts to separate out the different retinal ganglion cell types [3].

Identified clusters were then divided manually into putative ON, OFF, or ON-OFF categories, depending on the responses to the ON and OFF flash in the first 2 seconds of the chirp stimulus. As expected from previous studies on salamander, most cells showed either an OFF or ON-OFF response [14].

References

[1] Baden T, Berens P, Franke K, Román Rosón M, Bethge M, Euler T. The functional diversity of retinal ganglion cells in the mouse. Nature. 2016;529(7586):345-50.

[2] Trapani F, Spampinato GL, Yger P, Marre O. Differences in nonlinearities determine retinal cell types. Journal of Neurophysiology. 2023 Sep 1;130(3):706-18.

[3] Segev R, Puchalla J, Berry MJ. Functional organization of ganglion cells in the salamander retina. Journal of neurophysiology. 2006 Apr;95(4):2277-92.
